# Streets and Stress: A Pilot Study on How Quality and Design of Streets Impacts on Urban Stress

**DOI:** 10.1177/19375867231200584

**Published:** 2023-09-25

**Authors:** Fereshteh Sadeghpoor, Ehsan Ranjbar, Maryam Esmaeilinasab, Mir Hojjat Seyed Valiloo, Mark J. Nieuwenhuijsen

**Affiliations:** 1 Faculty of Art and Architecture, Tarbiat Modares University (TMU), Tehran, Iran; 2 Department of Architecture, Aalto University, Espoo, Finland; 3 Arsan Research Group, Tehran, Iran; 4 Department of Psychology, Tarbiat Modares University (TMU), Tehran, Iran; 5 Barcelona Institute for Global Health (ISGlobal), Spain

**Keywords:** urban space, stress, street type, therapeutic space, EEG

## Abstract

**Objectives:**

To identify how quality and design of streets impacts urban stress.

**Background:**

Few studies have comprehensively addressed environmental factors affecting stress in urban public spaces. However, a remarkable portion of our everyday life is spent in public spaces, particularly streets.

**Method:**

This study seeks to evaluate the effect of three types of streets as major public spaces on stress. These include a street with the dominance of green spaces (A), a motorist-oriented street (B), and a pedestrian street (C). For this purpose, we selected a group of participants (*n* = 16) aged 20-30, with an equal number of men and women who were generally healthy and had normal stress levels. Participants were asked to wear an electroencephalogram (EEG) headset, walk different streets, and answer the Perceived Restorativeness Scale (PRS) and urban design qualities questionnaires.

**Results:**

According to the results, participants experienced the highest stress in street type B and the lowest in type A.

**Conclusions:**

Green space and vegetation, a sense of security, privacy and coziness, climatic comfort, and safety of space had the most positive effect on stress reduction. Whereas noise pollution, vehicle traffic, and crowdedness were the most critical factors of stress. Finally, our findings suggest that the component of green space has a more significant effect on stress reduction compared with the elimination of vehicle traffic.

## Introduction

World Health Organization (WHO) has defined health as complete physical, mental, and social well-being beyond the mere absence of disease (WHO, 2020). These dimensions affect each other, and the importance of mental health is so great that health is impossible without the mental component. According to the World Health Report, one in four persons in the world experience neurological or psychological disorders sometime in their life. In 2019, 970 million people around the world were living with a mental disorder and the figure continues to rise as a result of increasing urbanization (Mccay et al., 2017). Biological disorders have always been more common in urban than in rural areas, and this has been historically associated with higher rates of psychological diseases such as depression and schizophrenia (Adli, 2011; Heinz et al., 2013; Hosang, 2016).

In today’s modern society, stress is one of the factors of health that potentially underlies many diseases (Grahan & Stigsdotter, 2003). Stress is essentially not harmful and can stimulate progress in an individual (Burton, 1990; Grahan & Stigsdotter, 2010). However, urban life prevents complete recovery from tensions and exposes humans to constant stress. Stress contributes between 70% and 80% to the occurrence or worsening of diseases and WHO has announced it as the most serious challenge of the 21st century (Fink, 2017).

This study seeks to measure the effect of the different dimensions of street design on citizens’ stress levels and present a novel perspective on how stress can be controlled in public urban spaces.

Various factors in public spaces, such as noise pollution, visual pollution, air pollution, crowdedness, social conflicts, decreased sense of belonging, scarcity of green space, and lack of security, tend to create psychological tensions and negatively affect people’s physical and mental health (Allahyari et al., 2017; Cutrona et al., 2006; Groenewegen et al., 2018; Gruebner et al., 2017; Halpern, 1995; Hematian & Ranjbar, 2022; Layeb et al., 2016; Schweitzer et al., 2004). These psychological tensions which lead to mental health disorders are called stress.

As a large part of our everyday life is spent in public urban spaces, their quality and design may directly impact stress levels. The literature on the relationship between public urban spaces and stress is still at its beginnings. Many studies in this field focus on the impact of exposure to natural versus built environments (Ulrich et al, 1991; Van den Berg et al., 2014; Wang et al., 2016) and much of the focus has been virtual or in a lab. In addition, we need holistic studies that investigate the effect of different dimensions of public spaces (physical, social, etc.) and combine the methods of assessing mental health with those of urban public spaces.

The majority of the studies of mental health in urban settings have been conducted in developed countries; however, many of the most stressful cities in the world are located in undeveloped or developing countries. It seems the issue of mental health is more serious in these countries. This study aimed to evaluate the urban stress in Tehran, a city in a developing country, Iran. According to the results of research by Zipjet Company in 2017, Tehran is named the 6th most stressful city in the world, which can be partly attributed to the quality of public urban spaces.

### Stress and Public Urban Spaces

Hans Selye first used the term “stress” in 1935 to explain tension factors in living creatures (Fink, 2017). Since then, stress studies have adopted various epidemiological, psychological, and biological approaches (Cohen et al., 2016). The epidemiological approach concerns stressors related to life events (Holmes & Rahe, 1967) and environmental and socioeconomic factors (Burton, 1990). Through a psychological approach, Lazarus and Folkman showed that the intensity of a stressor is not necessarily the same for all people (Cohen et al., 2016). Stress is a consequence of a lack of balance between an individual’s perception of environmental conditions and their assessment of their ability to respond to those conditions (Evans & Cohen, 2004), which shows the role of individual differences (Cohen et al., 2016; Godha, 2015; Grahan & Stigsdotter, 2003). The biological approach focuses on the human body’s changes in reaction to stressors. Increased heart rate, blood pressure, and cortisol levels are among the physiological symptoms that can lead to cardiovascular diseases, diabetes, and Alzheimer’s disease in the long term (Adli, 2011; Burton, 1990; Kondo et al., 2018). In the last 20 years, stress studies have tended to consider combining these approaches (Cohen et al., 2016).

The environment has historically been regarded as a factor that influences human health. In other words, our quality of life depends on the quality of the environment in which we live (Adli, 2011; Burton, 1990; Elsamahy & Abd El-Fattah, 2018; Evans & Cohen, 2004; Godha, 2015; Hansmann et al., 2007; Sarkar et al., 2013). Today, our environment is polluted with a large number of stressors. The first studies of environmental effects on stress focused on the natural environment. In 1979, Roger Ulrich tested the hypothesis that nature would contribute to reducing stress (Ulrich et al., 1991). Furthermore, many similar studies ensued at the beginning of the 1980s (Annerstedt et al., 2010). Almost all of these studies confirmed the positive effects of the natural environment on stress levels in comparison with the built environment (Gidlow et al., 2016; Groenewegen et al., 2018; Hartig et al., 2003; Hedblom et al., 2019; Kondo et al., 2018; Ulrich, 2002; Ulrich et al., 1991; Van den Berg et al., 2003; Wang et al., 2016). Living in nature reduces the body’s physiological response to stress by enhancing the function of the cardiovascular system, thereby improving one’s adaptability in the face of stressful events (Gascon et al., 2016; Groenewegen et al., 2018). The natural environment is not limited to green space; other natural elements may also have therapeutic power. Measuring cortisol levels in 20 women by listening to the sound of water, relaxing music, and silence showed that the sound of water is more effective in reducing cortisol levels (Thoma et al., 2018).

There are two theoretical explanations for restoration environments: First, stress recovery theory is concerned with restoration from stress, which occurs when an individual is confronted with a situation perceived as demanding or threatening to well-being. Ulrich’s theory proposes that natural environments promote recovery from stress, while urban environments tend to hinder the same process (Ulrich et al., 1991). Second, attention restoration theory (ART) focuses on restoring attentional fatigue after prolonged engagement in mentally fatiguing tasks. The Kaplans describe four components for the healing power of nature, namely, being away, fascination, extension, and compatibility, which attract human attention without wasting energy, help to relieve mental preoccupations, and reduce stress by reconstructing the mind and enhancement of mental capacity (Kaplan, 1995).

Although many stress and environmental studies have focused on green spaces, public urban spaces are not confined to green spaces. The typology of urban public spaces is a complicated issue that has been addressed by many scholars from the viewpoint of form, function, sociocultural, and socioeconomic aspects. The most prevalent method of typology is based on urban forms. Despite some theoretical differences, most proponents of this typology have recognized streets and squares as two major types of urban space. In urban design, the most crucial component of a successful public space is its qualities (both physical and nonphysical); being green is only one of these qualities. A glance at the literature in this field indicates that researchers have dealt with only several dimensions of public spaces that might affect stress. There seems to be a need for a comprehensive view of the factors of urban public spaces that influence stress levels. In this section, we shall attempt to analyze different urban stress factors and propose a comprehensive model of stress factors in urban spaces.

Research findings suggest that crowded environments likely increase tensions (Evans & Cohen, 2004; Halpern, 1995; Loo, 1977). In other words, crowding is a social stressor that increases people’s stress levels by limiting their ability to control their environment (Adli, 2011). If a design provides acceptable territory and privacy, the crowding and stress will be reduced (Loo, 1977).

Climate change is another issue that may directly or indirectly affect human mental health. Violence is more prevalent in warmer cities, and exposure to excessive heat increases secretion of stress hormones (Cianconi et al., 2020; Halpern, 1995; Mazlomi et al., 2017). The existence of green spaces and water-containing landscapes like canals will reduce heat island effects and stress (Gruebner et al., 2018). Tall structures intensify winds and reduce comfort, and cold or hot winds produce stress and anxiety (Halpern, 1995). Sometimes, the wind produces sounds that lead to stress (Brosschot et al., 2018). Climate change consists of many components whose effect on stress requires in-depth research.

Research into the effect of soundscape on human health has demonstrated that long-term exposure to traffic noise and other noise pollutions could increase stress hormones (Gruebner et al., 2017; Halpern, 1995; Layeb et al., 2016; Westman & Walters, 1981). On the other hand, natural sounds and relaxing music can calm the mind and reduce stress (Alvarsson et al., 2010; Linnemann et al., 2015; Thoma et al., 2013; Thoma et al., 2018).

Ninety-one percent of the world’s population is deprived of clean air (WHO, 2016). Studies suggest that negative personality traits are more prevalent among inhabitants of areas with polluted air. Smells are an important factor in air pollution (Halpern, 1995). Analysis of brain waves after smelling perfumes (Sowndhararajan & Kim, 2016) and research into the effects of smelling the fragrance of rose and lavender (Kianpour et al., 2016) have confirmed the positive influence of olfactory sense on the reduction of stress, whereas smelling unpleasant odors have been found to be a factor of creating fear, anxiety, and stress (Schweitzer et al., 2004).

In addition to noise and air pollution, the adverse effects of visual pollution on mental health have recently become the subject of research in this area (Yilmaz & Sagsoz, 2011). Visual pollution may lead to behavioral disorders and stress (Allahyari et al., 2017; Elsamahy & Abd El-Fattah, 2018). Various components such as color, brightness, green space, order, cleanness, and taking care of the space play a role in visual comfort (Allahyari et al., 2017).

Colors affect the secretion of hormones and, thus, contribute to different emotions, behaviors, and personality traits (Dargahi & Rajabnezhad, 2014). Colors with shorter wavelengths induce a sense of coolness and peace (Elliot, 2015). People experience more stress in a red room than green and white one (Kutchma, 2003). Color is an important component of art and in addition to the effect caused by colors, art styles can affect stress. some art styles such as abstract paintings that depict disorder and chaos may increase stress (Hassell, 2014), whereas images of warm seasons that display positive subjects, nature, and friendly relationships among people are more appropriate for therapeutic purposes (Schweitzer et al., 2004). In addition to color and artistic styles, the material used in the design and production of objects can influence human feelings (Crippa et al., 2019).

Another factor that affects stress is light and brightness (Godha, 2015). In contrast to artificial light, which induces a lousy mood and restlessness, natural light increases recognition of visual details and, therefore, better perception of information while at the same time affecting the perceived thermal comfort in an urban space (Münch et al., 2017; Schweitzer et al., 2004). Light is one of the essential components in urban design, which is related to the quality of a “sense of security” in the urban space.

Humans have an innate fear of the unknown. As they grow up, they get to know the signals of a sense of security. As long as an individual does not feel secure in an environment, their body is prepared to face any strange thing; but when the sense of security is felt, they achieve peace of mind, and the stress mechanism is deactivated (Brosschot et al., 2018). Fear of being victimized is the leading cause of stress, and the inhabitants of neighborhoods with high crime rates are likely to have higher stress levels (Cutrona et al., 2006). Environments with high visibility, a space’s capability of being seen entirely, the visibility of an individual from every point in the space, and decreased building surface can also reduce stress levels. However, motor vehicle traffic and the safety of pedestrians have a crucial role in perceived stress (Knoll et al., 2017). The existence of strong social networks and natural factors, maintenance of space according to the theory of broken windows, and legibility of the environment, are positive signs for inducing the sense of security and safety and decreasing stress and anxiety (Brosschot et al., 2018; Mccay et al., 2017; Tabatabaian & Tamannaee, 2014).

The human mind is more at peace when others are present. In other words, belonging to a group and receiving social support increases the chances of survival and achieving one’s goals and helps to relieve fear and anxiety (Brosschot et al., 2018; Ward Thompson et al., 2016). As humans are social creatures, membership in a group creates a sense of security. Social interaction can decrease crime rates in a neighborhood; therefore, children are better cared for, and residents feel happier about their surroundings (Jackson, 2003), which indicates the importance of attendance quality in stress reduction. It is essential to strike a balance between allowing for social interaction and connection, while also providing enough personal space and comfort to avoid feelings of crowding and anxiety.

Cohesion and integrity allow for a better perception of environmental stimuli, which could reduce tension (Knoll et al., 2017; Tabatabaian & Tamannaee, 2014). Legibility is one of the central concepts of cohesion. When space design is legible, one can perceive the components in an integrated framework without any information contradiction and ambiguity in mind, which causes more control over the environment and less stress (Tabatabaian & Tamannaee, 2014).

Sport and physical activity positively influence stress levels and one’s personality traits and self-esteem apart from duration and intensity, gender, age, and health status (Barton & Pretty, 2010; Fox, 1999; Paluska & Schwenk, 2000). Exercising, bike-riding, and other sports activities in green spaces are more effective in reducing anxiety than light activities such as walking and resting (Hansmann et al., 2007). Active urban space design motivates physical activity. Pedestrian-oriented spaces encourage people to walk and perform physical activity and provide an opportunity for social interaction and encountering nature (Mccay et al., 2017).

Urban environments with a mix of land uses (e.g., residential, commercial, and recreational) are associated with lower levels of psychological distress and improved mental health outcomes, compared to environments with a more segregated land-use pattern (Sarkar et al., 2013). Land use is a significant factor in physical activity and outdoor recreation, particularly among those with lower economic status who engage in physical activity primarily for transportation purposes rather than recreation. Land-use planning can affect air and noise pollution, and certain land uses, such as a church, may reduce exposure to violence and increase social support (WHO, 2010).

Living on the upper floors of buildings is associated with less physical activity, behavioral problems, respiratory diseases in children, neurosis, and social isolation (Jackson, 2003). People living in areas with higher density are 67%-88% more likely to be diagnosed with mental disorders and 12%-20% more likely to develop depression (Moughtin et al., 2009; Sundquist et al., 2004). Apart from the type of land use, high-rise buildings have a repressive effect on pedestrians and may increase anxiety. This effect can be mitigated by using shades in the street that could partially block these buildings from people’s view (Gruebner et al., 2017).

The scale of space can also influence mental health. Large-scale squares and streets with less complexity of details in the facades are likely to increase stress, and this is an emphasis on the advice of planners and architects such as Jan Gehl, who are opponents of building less-detailed spaces with nonhuman scales (Knoll et al., 2017). The larger size of the public open space near a residential place will increase the inhabitants’ amount of walking (Koohsari et al., 2018).

As mentioned before, the ability to control space affects stress reduction, and this is precisely the definition of flexibility quality in urban design. When you need to change the space and face limitations, you will gradually experience psychological reactions such as stress, anger and aggressiveness, helplessness, and ultimately, disappointment. Flexibility can help to reduce stress by creating spaces that are more adaptable and responsive to the needs of individuals (Tabatabaian & Tamannaee, 2014). When individuals encounter obstacles or movement limitations such as excessive street slopes in a neighborhood that can make it difficult for them to move around and access their needs, they may experience psychological distress (Sakar et al., 2013).

Given the inverse correlation between happiness and perceived stress (Schiffrin & Nelson, 2010), it seems that creating happy urban spaces may help to reduce the users’ stress. The physical features of space, including environmental elements, pedestrian-orientedness, and cohesion and integrity, are the most critical indicators of achieving happiness, liveliness, a sense of security, and nostalgia could increase happiness levels in a space (Samavati & Ranjbar, 2017).

As discussed above, each study in the literature has mentioned one or more stress factors, which are summarized in Table 1.

**Table1.** Factors Affecting Stress.

Factors Affecting Stress		Studies
Sound	Noise pollution	WHO (2011); Layeb et al. (2016); Ulrich (2002); Evans & Cohen (2004); Westman & Walters (1981); Halpern (1995); Adli (2011); Gruebner et al. (2017); HASSELL (2014); Burton (1990); Alvarsson et al. (2010); Thoma et al. (2013); Linnemann et al. (2015); Thoma et al. (2018); Schäfer et al. (2015); and Rishi & Khuntia (2012)
	Music (soft natural sounds, etc.)	Ulrich (2002); Schweitzer et al. (2004); and Thoma et al. (2018)
Air	Air pollution	Halpern (1995); Schweitzer et al. (2004); Adli (2011); Gruebner et al. (2017); P. Groenewegen (2018); HASSELL (2014); Burton (1990); and Rishi & Khuntia (2012)
	Smell	Schweitzer et al. (2004); Sowndhararajan & Kim (2016); and Kianpour et al. (2016)
Visual components	Legibility (lack of complexity and mysteriousness, lack of ambiguity)	Kaplan & Kaplan (1982); Ulrich (2002); HASSELL (2014); Jackson (2003); and Tabatabaian & Tamannaee (2014)
	(Disorder, lack of maintenance)	Knoll et al. (2017); HASSELL (2014); Allahyari et al. (2017); Elsamahy & Abd El-Fattah (2018); and McCay et al. (2017)
Use of local and natural materials		Wang et al. (2016) and Crippa et al. (2019)
Light and illumination		Hill (2008); Schweitzer et al. (2004); HASSELL (2014); Godha (2015); Münch et al. (2017); and Ghanbaran et al. (2018)
Color		HASSELL (2014); Hill (2008); Schweitzer et al. (2004); Dargahi & Rajabnezhad (2014); Kutchma (2003); Yoto et al. (2007); and Elliot (2015)
Art		Schweitzer et al. (2004); HASSELL (2014); and Ulrich (2002)
Natural areas and green space		Wang et al. (2016); Hartig et al. (2003); Ulrich et al. (1991); Ulrich (2002); Van den Berg et al. (2003); Gidlow et al. (2016); Kondo et al. (2018); Groenewegen et al. (2018); Hedblom et al. (2019); Ward Thompson et al. (2012); Ward Thompson et al. (2016); Kaplan and Kaplan (1989); Gascon et al. (2016); Annerstedt et al. (2010); Roe et al. (2013); Moughtin et al. (2009); HASSELL (2014); Burton (1990); and Parsons et al. (1998)
Social interaction and support		Halpern (1995); Ward Thompson et al. (2016); Ulrich et al. (2004); Gruebner et al. (2017); Cutrona et al. (2006); HASSELL (2014); Jackson (2003); Burton (1990); Brosschot et al. (2018); and Rishi & Khuntia (2012)
Security		Halpern (1995); Knoll et al. (2017); Ulrich (2002); Groenewegen et al. (2018); Cutrona et al. (2006); Gruebner et al. (2017); Layeb et al. (2016); Burton (1990); and Brosschot et al. (2018)
Crowding		Loo (1977); Halpern (1995); Evans & Cohen (2004); Adli (2011); Ulrich (2002); and HASSELL (2014)
Climate	Temperature	Cianconi et al. (2020); Halpern (1995); and Mazlomi et al. (2017)
	Wind	Halpern (1995) and Brosschot et al. (2018)
Height of buildings		Sundquist et al. (2004); Knoll et al. (2017); Gruebner et al. (2017); Jackson (2003); and Moughtin et al. (2009)
Land use		Sarkar et al. (2013); WHO (2010); Cutrona et al. (2006); and Jackson (2003)
Physical activity and sport		Barton & Pretty (2010); Fox (1999); Hansmann et al. (2007); Paluska & Schwenk (2000); Ward Thompson et al. (2016); Jackson (2003); Burton (1990); and McCay et al. (2017)
Scale		Knoll et al. (2017) and Koohsari et al. (2018)
Safety		Halpern (1995); Layeb et al. (2016); and Knoll et al. (2017)
Flexibility		Hartig et al. (1997) and Tabatabaian & Tamannaee (2014)
Obstacles and limitations of design (lack of inclusiveness)		Layeb et al. (2016) and Sarkar et al. (2013)

**Figure 1.**
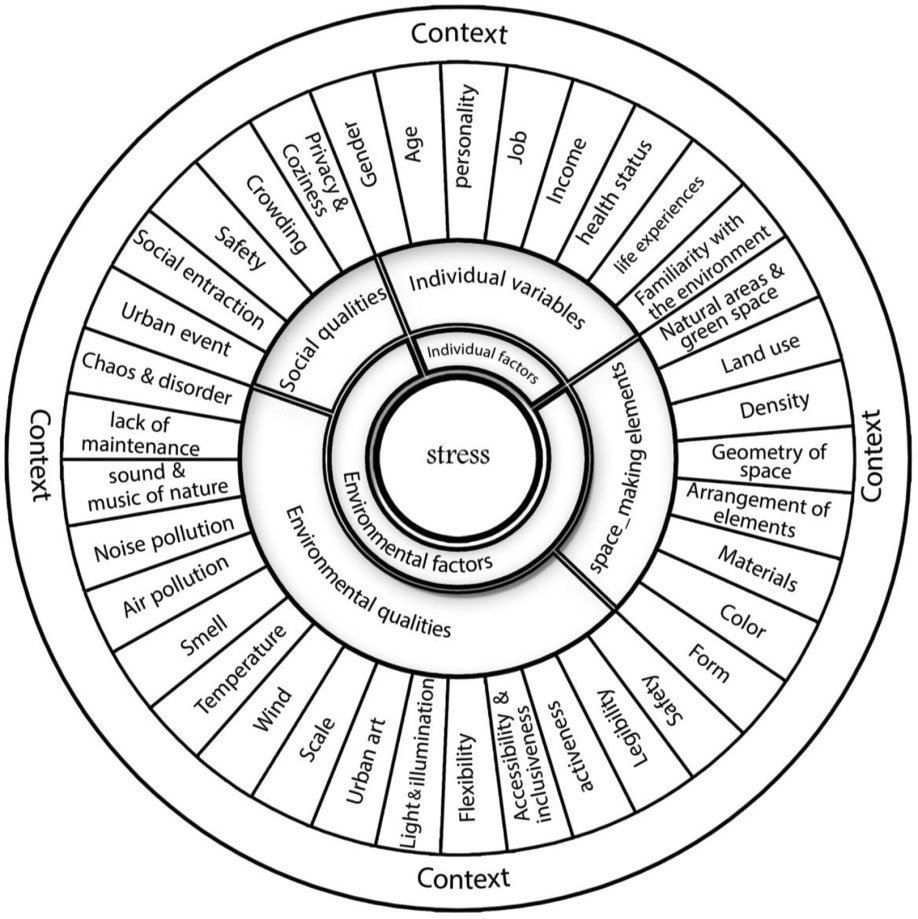
The conceptual model.

An analysis of the above items would disclose that the main stress factors are personal and environmental. Environmental factors can be divided into three categories: space-creating elements, social quality, and environmental quality. Figure 1 depicts a comprehensive model of stress factors in public spaces.

The abovementioned studies have used various instruments for measuring stress depending on the nature of the studies. The most common method is to use self-report questionnaires such as Holmes and Rahe Stress Scale, Cohen Stress Scale (Hellhammer et al., 2010), and the Depression Anxiety Stress Scales (DASS) questionnaire (Lovibond & Lovibond, 1995). A review of these questionnaires shows that they mainly measure the stress coming from different aspects of a person’s life rather than the stress perceived through environmental effects. Some studies have used the PRS questionnaire to measure the healing effect of the environment (Hartig et al., 1997; Wang et al., 2016). Based on the body’s reactions to stressful situations, another group of studies has attempted to evaluate stress by measuring cortisol levels (Roe et al., 2013), measuring skin conductivity (Layeb et al., 2016), and performing electrocardiogram (Wang et al., 2016) and EEG tests (Aspinall et al., 2015). More recent studies have combined several methods to increase the accuracy and validity of the results (Thompson et al., 2012; Wang et al., 2016).

### Knowledge Gap

Much of the focus on gray versus green has been virtual while real-world experience is quite different and much more complicated. This study seeks to identify and use a proper combination of the existing instruments to evaluate environmental stressors within the real environment and determine which type of urban street is most effective in reducing stress levels.

## Method

This research is a cross-sectional study, and the possibility of change in chronic stress is very low in this type of study. Therefore, acute stress and perceived stress have been emphasized.

### Participants

As a pilot study and due to financial constraints, we used a sample of 16 participants. To homogenize the sample and control the effect of individual factors (such as age, gender, and educational status), we selected a group of students in the age range of 20-30 years, with an equal number of men and women. We invited Tarbiat Modares University’s students to participate in the test. Considering the effect of health status and stress levels on the test results to assess the general health status of the participants, they were asked to complete the General Health Questionnaire (GHQ). The GHQ is a self-report screening tool used to detect possible psychological disorders (Goldberg & Hillier, 1979). Stress status was also monitored using the DASS-21 questionnaire. The DASS-21 is a self-report scale designed to measure the negative emotional states of depression, anxiety, and stress (Lovibond & Lovibond, 1995). Based on the results of these two questionnaires, of 25 participants, 16 made it to the next stage.

### Experimental Design and Procedures

Each participant was asked to wear the EEG device and walk over the specified path while the research team followed them within a distance of 8-10 m so that they would not affect the participant’s behavior. The team exactly recorded their actions and reactions. After the walk, they were asked to fill out two questionnaires, that is, PRS and the questionnaire for evaluating the environmental qualities affecting stress. Each participant walked on three designated streets (A, B, and C), and the attendance order on the streets was the same for all participants.

We performed our experiments in the middle of the week to ensure that people were doing their everyday activities and tried to minimize the varying effect of time on the participants’ experience by performing the experiments at a fixed time in the afternoon in July.

#### Site selection and categorization

**Figure 2.**
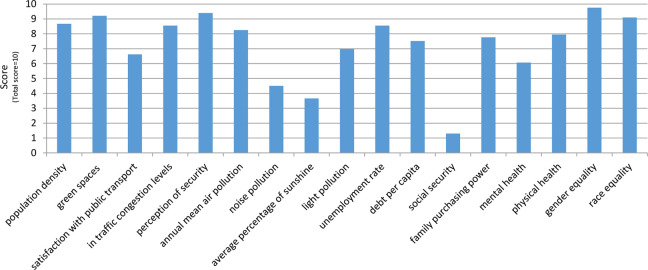
Factors of stress in Tehran. *Source*: https://financialtribune.com/node/72456.

Ranking of stressors in Tehran indicates that air pollution, noise pollution, and traffic are among the most important environmental stressors (Hataf et al., 2015). According to the results obtained by Zipjet Company in 2017, Tehran ranks sixth among 150 cities in terms of stress level. Factors of stress in Tehran have been evaluated in Figure 2.

Given the city’s status in comparison with other cities of the country, this study focused on the physical-functional center of Tehran, which is representative of its different districts and has the highest density of people’s presence. After examining the typology of streets, the most important streets in terms of stress factors were identified.

**Figure 3.**
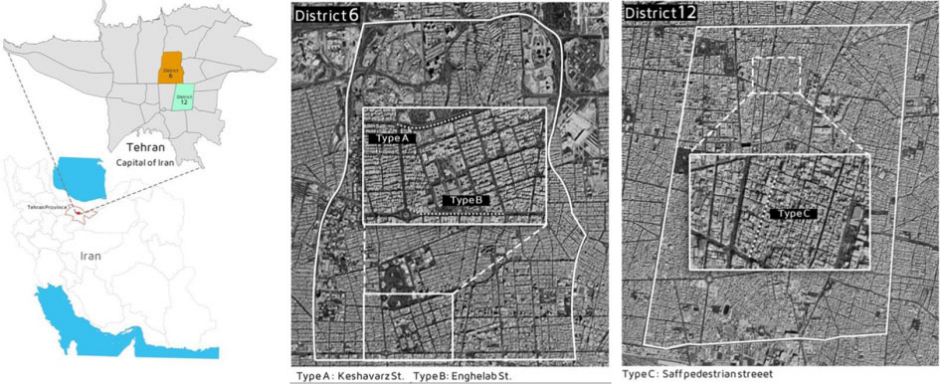
The location of the selected streets.

The street types selected in this study include a street with green space and appropriate sidewalks (Type A), a car-dominated street (Type B), and a pedestrian street (Type C). An equal stretch of the three streets was specified so that the participants could spend equal time in all three streets (disregarding their speed). Figure 3 shows the location of the three selected streets.

##### Type A

**Figure 4.**
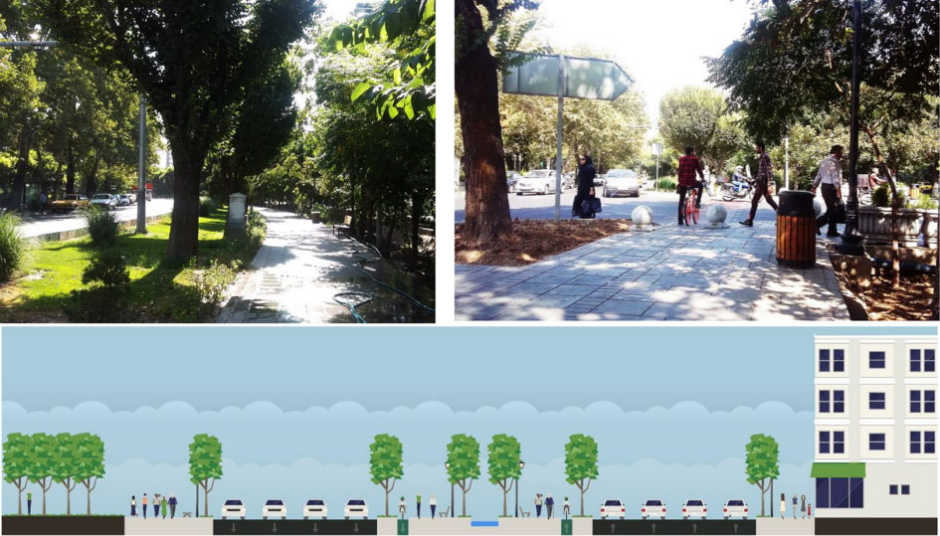
Type A: Keshavarz Street (cross-section and photos).

It is one of the most popular streets in Tehran, which has good recreational facilities. The central lane of this street is a green path for pedestrians and bikers. The park, located on the edge of this street, contributes to the area’s visual beauty and air quality. The water canal inside the central green pedestrian path and the chanting birds are invaluable features that are quite attractive to users (see Figure 4).

##### Type B

**Figure 5.**
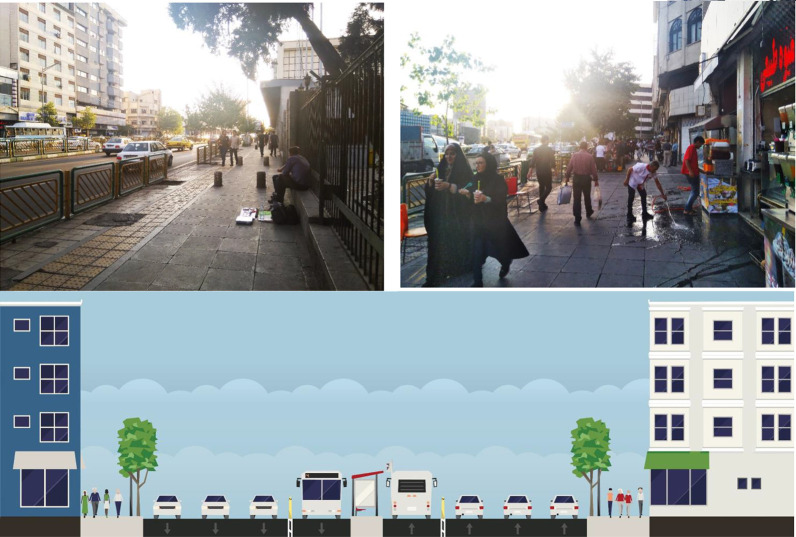
Type B: Enghelab Street (cross-section and photos).

As one of the main central streets of Tehran, this street acts as a hub in the communication network and has a high level of traffic and noise pollution. The concentration of different commercial and administrative uses, book publishers, and the university attracts many people from all over the city (see Figure 5).

##### Type C

**Figure 6.**
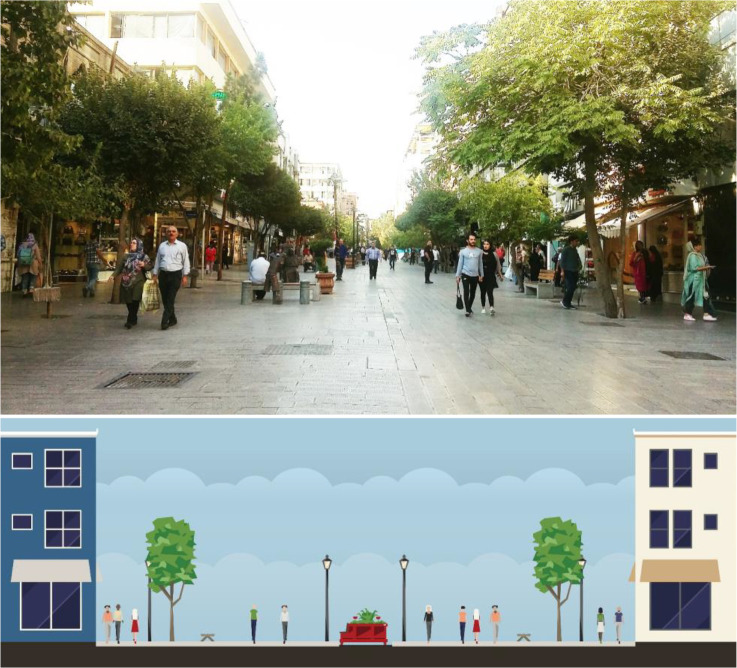
Type C: Saf Pedestrian Street (cross-section and photos).

This pedestrian street is a historical axis that is now a completely pedestrian area with considerable tranquility. It is famous for women’s bags, shoe stores, and other appealing uses such as floristry and restaurant (see Figure 6).

### EEG

**Figure 7.**
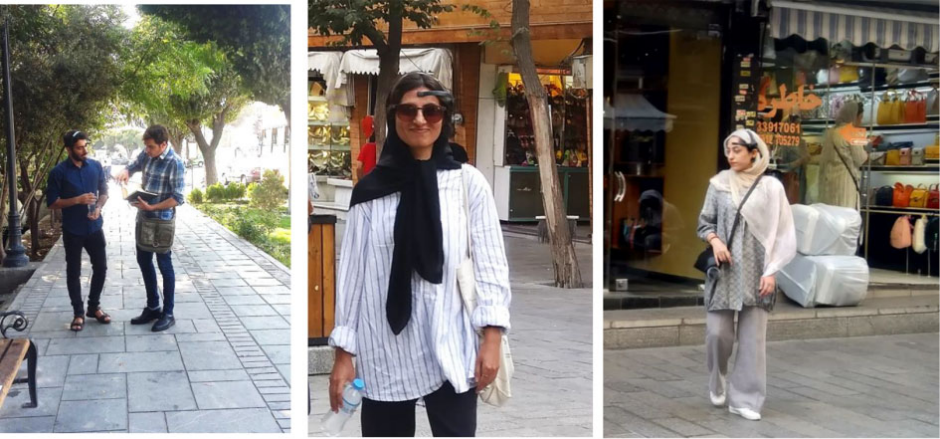
Photos of participants with EEG device.

**Figure 8.**
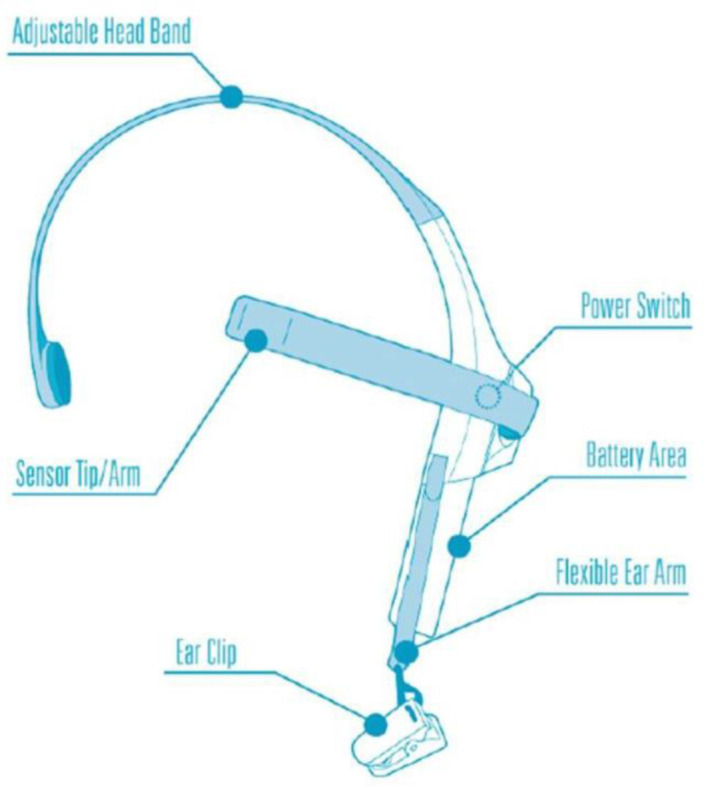
The structure of the single-channel EEG device. *Source*: Hareendar et al. (2019, p. 156).

**Figure 9.**
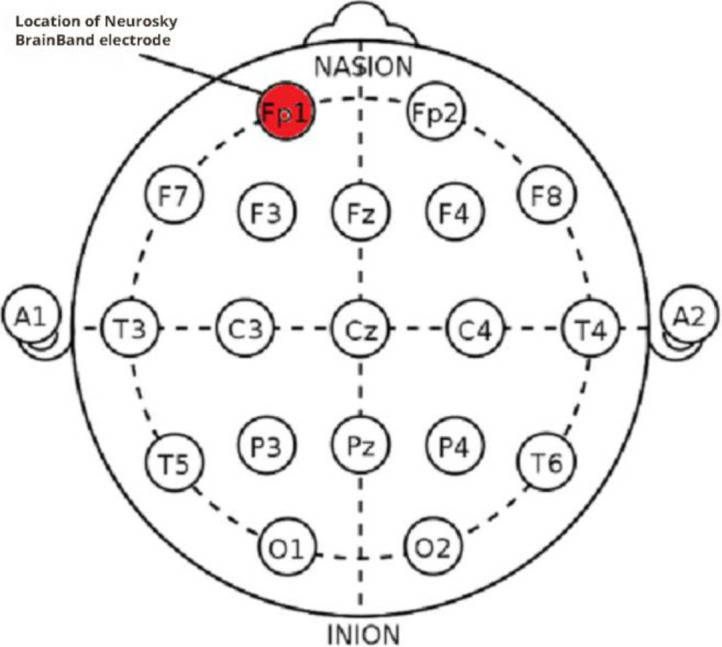
Electrode position. *Source*: Hareendar et al. (2019, p. 156).

Due to the lack of access to portable multichannel EEG, we were confined to using single-channel EEG devices. We used a Neurosky single-channel EEG device in our experiments. Despite its relatively low price, its efficiency in recording the user’s mental status has been validated (Lim & Chong Chia, 2015). The accuracy of the device to detect emotions has been recorded at 81% (Quesada-Tabares et al., 2017). Figure 7 shows participants wearing the device on their heads. The device is first worn on the head. Next, it is clipped onto the ear, and the electrode is put on the forehead (see Figures 8 and 9). After turning it on, it is connected via Bluetooth to a smart device. When the electrode is located correctly, a green light is turned on, and the start button is activated. The participant then begins to walk over the determined path. The software installed on the smart device records and shows the participant’s mean level of relaxation.

### Measuring the Environment’s Healing Potential by PRS Questionnaire

The PRS questionnaire developed by Hartig et al. (1997) is based on ART theory and has been widely used as an instrument for measuring the healing quality of the physical environment (Hartig et al., 1997; Wang et al., 2016, p. 116). This questionnaire contains four components: being away, extension, fascination, and compatibility. In this study, we utilized the 26-item version of the PRS questionnaire. The subjects consider their personal experience of the environment and express their agreement on a 7-point Likert-type scale from 1 (*totally disagree*) to 7 (*totally agree*).

The score of the questions that measure the environment’s negative effects is calculated inversely, and the final score is calculated. A higher score indicates a higher healing potential. The reliability of the questionnaire was confirmed through a Cronbach’s α value of .82.

### The Questionnaire for Evaluating the Environmental Qualities Affecting Stress

**Figure 10.**
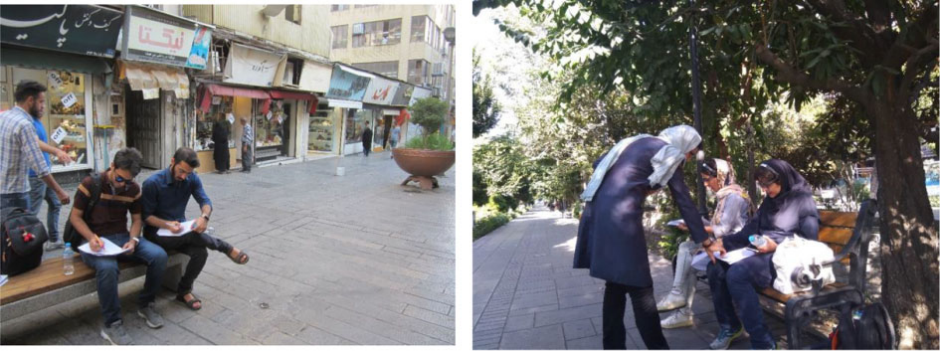
Participants filling out the questionnaire.

The questionnaire items were developed based on the conceptual model derived from the theoretical background. It should be noted that due to limitations in selecting the street types, we ignored some of the qualities included in the model. For example, as urban events were not performed in the selected streets, we did not incorporate this item in the questionnaire or, as this study was conducted during the day, the illumination factor was not included in the questionnaire. However, researchers have provided explanations about these qualities in the discussion section based on field observations. This questionnaire consisted of 17 items on a 7-point Likert-type scale ranging from 1 (*never*) to 7 (*completely*). For example, participants were asked to rate questions such as “How safe do you feel on this street?” To what extent do the heights of the buildings on this street make you feel relaxed? (see Figure 10) The face validity of this questionnaire has been determined by experts and its reliability with Cronbach’s α of .85. Finally, to calculate the total score, those items that measured the environment’s negative effect were calculated inversely. A higher score indicated an environment with a lower stress level.

### Data Analysis

The questionnaire data were analyzed in SPSS Version 25. The significance of the results was evaluated via the Friedman test and *t* test. The level of significance was determined as 0.05. The data recorded by the EEG device were converted to charts by the MATLAB software package.

## Results

### EEG Results

**Figure 11.**
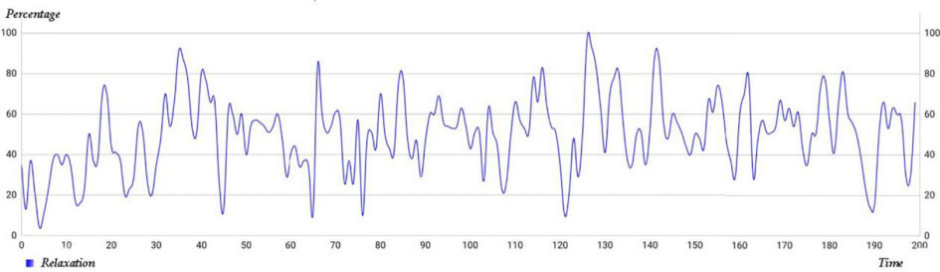
A sample of recorded EEG signals.

Figure 11 illustrates a sample of the signals recorded from one participant. At the end of the walk, the software (as mentioned in the Method section) displays the participant’s relaxation mean throughout the path.

Table 2 shows the highest and lowest numbers recorded as well as the mean relaxation level for each street type. The mean relaxation levels of streets were 48.56 for Type A, 46.13 for Type B, and 50.69 for Type C.

**Table 2.** Mean Difference of Street Type’s Electroencephalogram Signals.

Descriptive Statistics					
Streets Types	*N*	Minimum	Maximum	Mean	Standard Deviation
A (green)	16	28	62	48.56	9.128
B (car-oriented)	16	32	60	46.13	6.879
C (pedestrian)	16	37	62	50.69	8.039

A paired samples *t* test (Table 3) is used to compare the mean relaxation levels of the participants in each of street types (A, B, and C). The results shows the participants’ relaxation levels did not significantly differ between the three types of streets.

**Table 3.** Paired Samples *t* Test for Electroencephalogram Signals.

Paired Samples Test								
		Paired Differences						
		Mean	Standard Deviation	Standard Error Mean	95% Confidence Interval of the Difference	*t*	*df*	Sig. (Two-Tailed)
					[Upper, Lower]			
Pair 1	A-B^a^	−2.438	8.862	2.215	[2.285, −7.160]	−1.100	15	.289
Pair 2	B-C	−4.563	12.193	3.048	[1.935, −11.060]	−1.497	15	.155
Pair 3	C-A	2.125	12.811	3.203	[8.951, −4.701]	0.664	15	.517

^*a*^ Street types: A (green), B (car-oriented) & C (pedestrian).

### The Results of the PRS Questionnaire

The PRS questionnaire is used to evaluate the effect of each street type on the relaxation and mental improvement of the participants, with a central focus on the psychological dimension of the topic. Table 4 illustrates the mean values for both overall perceived restorativeness and each subscale.

**Table 4.** Means and Standard Deviations for Perceived Restorativeness Scale (PRS) and Subscales.

Type of Street	Overall PRS (Perceived Restorativeness)	Subscales			
		Being Away	Fascination	Coherence	Compatibility
	Mean (*SD*)	Mean (*SD*)	Mean (*SD*)	Mean (*SD*)	Mean (*SD*)
A (green)	125.75 (24.10)	22.38 (6.63)	37.62 (9.38)	22.62 (4.11)	43.13 (8.65)
B (car-oriented)	87.38 (16.82)	12.19 (4.88)	28.44 (8.62)	14.44 (4.48)	32.31 (4.52)
C (pedestrian)	102.69 (25.27)	17.94 (5.87)	33.00 (11.14)	19.37 (5.08)	32.38 (8.96)

*Note*. PRS based on 7-point scale, where lower values indicate lower levels of restorative experience. *N* = 16 for each street.

The Friedman test revealed a statistically significant difference between the mean values (*p* < .001) as presented in Table 5. According to Table 4, results indicate that Type A (125.75 ± 24.10) has the highest healing potential among the three types, with Type C (102.69 ± 25.27, *p* = .005) and Type B (87.38 ± 16.82, *p* = .001) ranked second and third, respectively.

**Table 5.** Overall Perceived Restorativeness Scale (PRS) Friedman Test.

Mean Difference	Keshavarz (A)	Enghelab (B)	Saf (C)	Overall PRS Friedman Test	
				Mean Rank	Test Statistics
A (green)	—	38.37***	23.06**	2.75	Chi-square = 15.77; *df* = 2; *p* < .001
B (car-oriented)		—	15.31*	1.41	
C (pedestrian)			—	1.84	

* *p* < 0.05

** *p* ≤ 0.01

*** *p* ≤ 0.001.

**Figure 12.**
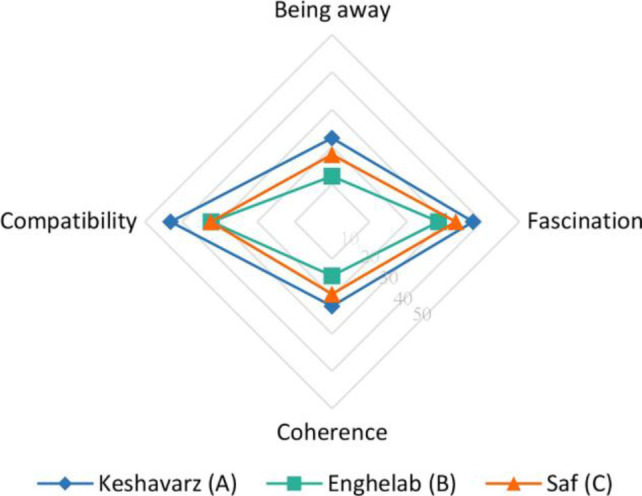
Comparison of Perceived Restorativeness subscales’ mean values.

The mean values on the subscales of being away, fascination, coherence, and compatibility are presented in Table 4. The results indicate a significant difference between the streets in each of the subscales (being away: *p* < .001; fascination: *p* = .05; coherence: *p* < .001; and compatibility: *p* = .001). According to Figure 12, Type A has the highest healing potential in each subscale, followed by Type C and Type B in sequence.

### The Results of the Questionnaire for Evaluating the Environmental Qualities Affecting Stress

The reliability of the results from each street is assessed by calculating Cronbach’s α using SPSS software. The resulting Cronbach’s α values for Type A, Type B, and Type C streets are .79, .81, and .80, respectively, indicating good internal consistency of the questionnaire responses in each street type (a Cronbach’s α value of .7 or higher is generally considered acceptable for research purposes).

**Figure 13.**
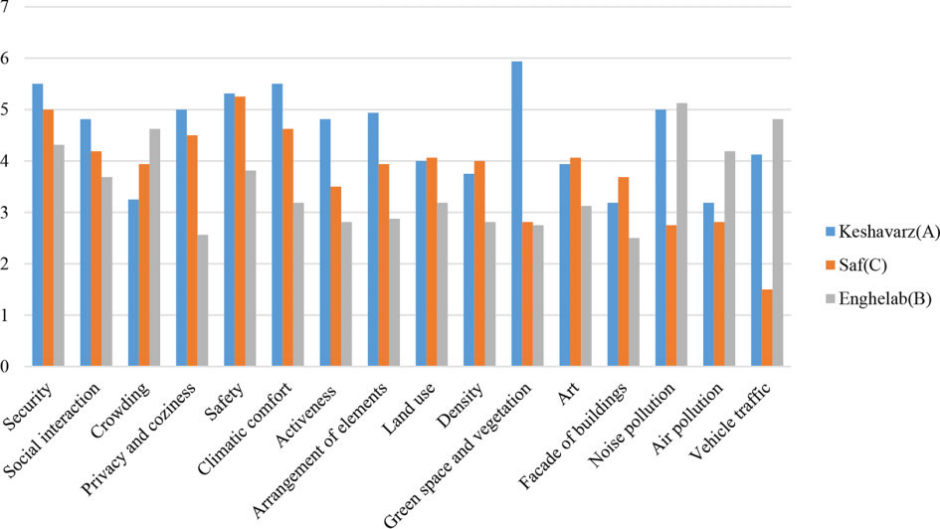
Mean value of the qualities in each street type.

According to the study, the mean values of participants’ responses for each quality are presented in Figure 13. Higher mean values indicate a greater positive effect on relaxation, excluding qualities related to noise pollution, crowding, vehicle traffic, and air pollution. For instance, the green space on Keshavarz Boulevard (Type A) has a greater positive effect on participants’ relaxation levels than the other two streets, while vehicle traffic on Enghelab Street (Type B) has a more negative effect.

The positive impact of different street types on participants’ relaxation, as measured by the mean values, is presented in Table 6. Statistical analysis using the Friedman test revealed a significant difference between these means, with a *p* value of .001. To further investigate these differences, pairwise comparisons were performed using the Wilcoxon test (Table 7). The Wilcoxon test results and mean values indicate that Type B has a significantly less positive effect on relaxation than the other two types. Additionally, Types A and C may have similar positive effects on relaxation. For the second time, Friedman’s test examined the significance of the difference between street types based on each quality separately. The results suggest that the level of relaxation is not significantly affected by the quality of social interaction (*p* = .06), art (*p* = .2), land use (*p* = .097), density (*p* = .94), or building facades (*p* = .14) across the different street types.

**Table 6.** Mean Values of Street Types' Positive Impact on Relaxation and Friedman Test.

Type of street	Mean (*SD*)	Friedman Test	
		Mean Rank	Test Statistics
A (green)	78.18 (10.38)	2.44	Chi-square = 22.95; *df* =2; *p* < .001
B (car-oriented)	54.25 (11.32)	1.03	
C (pedestrian)	75.18 (10.08)	2.53	

**Table 7.** Pairwise Comparisons by the Wilcoxon Test.

Mean Difference	Keshavarz (A)	Enghelab (B)	Saf (C)
A (green)	—	23.93***	3
B (car-oriented)		—	20.93***
C (pedestrian)			—

* *p* < 0.05. **p ≤ 0.01. ***p ≤ 0.001.

A pairwise comparison of the mean difference for each quality in street types by the Wilcoxon test is presented in Table 8. Numbers with asterisks in the statistical results indicate a significant difference between the two streets in relation to the specified quality. According to the results obtained in this table and Figure 13, it is possible to understand which streets have a significant difference in relation to each measured quality, and how they differ. For instance, participants believe that the perceived level of security in different types of streets varies significantly from one another (as indicated in the first row of Table 8). Type A street has the highest sense of security, followed by Type C and Type B streets, respectively (as shown in Figure 13).

**Table 8.** Pairwise Comparison of the Mean Difference for Each Quality in Street Types by the Wilcoxon Test.

Mean Difference Qualities	Keshavarz-Enghelab (A-B)	Keshavarz-Saf (A-C)	Saf-Enghelab (C-B)
Security	1.19***	0.50*	0.69*
Social interaction	1.12**	0.62	0.50
Crowding	1.38*	0.69	0.69
Privacy and coziness	2.44***	0.5	1.94***
Safety	1.5**	0.06	1.44**
Climatic comfort	2.31***	0.87	1.44*
Activeness	2.00**	1.31*	0.69
Arrangement of elements	2.06***	1.00*	1.06**
Land use	0.81	0.06	0.87*
Density	0.94	0.25	1.19*
Green space and vegetation	3.19***	3.13***	0.06
Art	0.81	0.12	0.93*
Facade of buildings	0.69	0.50	1.19*
Noise pollution	0.12	2.25***	3.63***
Air pollution	1.00*	0.38	1.38**
Vehicle traffic	0.69	2.62***	3.31***

*p < .05. **p ≤ .01. ***p ≤ .001.

## Discussion

This study investigates the provocation of stress in different urban street types and the factors involved. In contrast to previous studies (Neale et al., 2020; Wang et al., 2016), the participants were fixed to eliminate the effect of the personal variable and draw conclusions about the differences in street types with a higher confidence level. The results show higher levels of stress in the busy urban street compared with both green and pedestrian, the importance of using the right set of methods, the positive effect of green space, and vegetation of the street on reducing the negative effects of stressors and also shows what the most important qualities of low-stress urban spaces are.

The participants believed that heavy car traffic, noise pollution, crowding, and air pollution in Street B were stressful, and this has also been confirmed by previous investigations (Evans & Cohen, 2004; Gruebner et al., 2017; Neale et al., 2020; Schweitzer et al., 2004; Wang et al., 2016).

According to the questionnaire results for evaluating the environmental qualities affecting stress, there is no significant difference between Types A and C, whereas the PRS questionnaire indicates that Type A has a more significant potential for relaxation. First, this highlights the importance of methodology in the studies. If we examine the subjects’ selection among the three street types in three methods (quality evaluation, PRS, and EEG), we can find participants whose ratings turn out to be different through each method (e.g., Type A in quality evaluation, Type B in PRS, and Type C in EEG). Therefore, to measure environmental stress, it is necessary to study the environmental perception process and the functions of the brain because it may be the case that a factor neglected in the questionnaire is of greater importance to the mind.

***...it is necessary to study the environmental perception process and the functions of the brain because it may be the case that a factor neglected in the questionnaire is of greater importance to the mind***.

Using multiple methods for environmental quality evaluation is often neglected in majority of studies (Gidlow et al., 2016; Hansmann et al., 2007; Neale et al., 2020; Wang et al., 2016), but our study uses the Questionnaire for Evaluating the Environmental Qualities Affecting Stress along with other methods.

Second, given the characteristic feature of each street type (i.e., the rich natural features of Type A and the elimination of vehicle traffic in Type C), these two types are not equal. Thus, the lack of significant difference between these types seems to be because the components were not weighted. The question is: Which components are more effective in reducing stress? This highlights the importance of studying how influential factors affect each other. From the PRS results of these two street types (A and C), we can conclude that natural elements are more effective in stress reduction, and this is in line with previous findings (Hedblom et al., 2019; Gidlow et al., 2016; Groenewegen et al., 2018; Kondo et al., 2018; Neale et al., 2020; Wang et al., 2016).

In contrast to some of the previous studies (Neale et al., 2020; Wang et al., 2016), this study has measured the intensity of the effect of green space on other factors, such as traffic. As indicated by the participants’ responses, Type A is not a solely green street, and its car traffic, as well as noise pollution, is not significantly different from that of Type B; however, it provokes less stress than Type B and has a higher healing potential based on the PRS results, which means that the green space component seems to have reinforced the positive effect of other components and mitigated the negative components. We should also notice the effect of green space on the activeness of the environment. Although we expected the activeness of Type C to be highest due to its pedestrian-orientedness and lack of motor vehicle traffic, Type A was more active than the other two types.

Therefore, it can be argued that green space and vegetation play a more critical role in encouraging people to perform physical activity. Type B provoked the highest stress levels because of lower environmental qualities, notably noise pollution and car traffic. We also expected that eliminating motor vehicles from the urban space (Type C) would play a key role in reducing stress, but the results obtained for Type A suggested that this measure alone was insufficient. Previous studies in the literature mainly focused on a single quality, while the effectiveness of space results from an entire group of qualities. Among the qualities surveyed in this study, green space and vegetation, a sense of security, privacy and coziness, climatic comfort, and safety of space were found to have the strongest positive effect on the reduction of stress, while noise pollution, vehicle traffic, and crowdedness were the most important factors of stress among the users of space.

Examining the brain signals of the participants did not show any significant difference among the three street types, which may be explained by their anxiety about facing the EEG device, the small size of the sample, the nostalgic sense of the space for some participants, and the participant’s engagement with their mental preoccupations during the experiments. As with the sense of nostalgia, the two participants’ brain signals in Street B differed from the others and indicated a relatively more pleasant feeling. Interviews with these two participants revealed they had positive memories and experiences of this space. Good memories in space could probably counter the effect of negative components and reduce mental tension. In other words, it seems that the person’s perceived quality was different from that of the real space in a way that could reduce the deficiencies of the environment.

Conversely, a person may not perceive the advantages of a desirable space for some reason. For example, four participants had weaker brain signals in Street A. When interviewed, two of them stated that a serious preoccupation had occurred to them just before taking part in the experiments, and they had been busy thinking about the issue during the walk. However, they had high scores for Type A in their questionnaire.

Examining the level of EEG when the participants cross the intersections in Types A and B shows a decrease in relaxation. Another example is a flower store with a colorful showcase in Type C. In more than half of the subjects, the level of relaxation increased when watching the store. However, a more definite conclusion in this regard would require further research.

## Conclusion

Most studies on the relationship between environment and stress have only addressed one stressor, often virtually or in the lab. Among the few studies conducted in the real environment, this study addressed, for the first time, a set of factors that could affect stress. For this purpose, 16 students (an equal number of men and women) aged 20-30 with normal health and stress levels were selected as participants. They wore an EEG device and walked on three street types. After each walk, they completed two questionnaires.

The questionnaires’ findings suggest that green space and vegetation, a sense of security, privacy and coziness, climatic comfort, and safety of space were found to have the strongest positive effect on the reduction of stress, while noise pollution, vehicle traffic, and crowdedness were the most important factors of stress among the users of space. A comparison of the results concerning the street types indicates that although vehicle traffic, noise pollution, and the resulting sense of crowding increase stress levels, the quality of green space and vegetation in a street could mitigate these negative effects and even enhance positive components such as security in a way that the space might bear a generally positive effect on its users.

***...green space and vegetation, a sense of security, privacy and coziness, climatic comfort, and safety of space were found to have the strongest positive effect on the reduction of stress, while noise pollution, vehicle traffic, and crowdedness were the most important factors of stress among the users of space***.

Appropriate vegetation to create pleasant colors and smells, using vegetation as acoustic insulation and safety walls for pedestrians, use of water in the design of pedestrian paths, focus on the climatic comfort of pedestrians, creating comfortable isolated spaces, and special design for intersections of pedestrian and vehicle paths are among the most important design features of shared streets that could help to produce less stressful spaces.

***Appropriate vegetation to create pleasant colors and smells, using vegetation as acoustic insulation and safety walls for pedestrians, use of water in the design of pedestrian paths, focus on the climatic comfort of pedestrians, creating comfortable isolated spaces, and special design for intersections of pedestrian and vehicle paths are among the most important design features of shared streets that could help to produce less stressful spaces***.

Decisive conclusions about the effects of pedestrian streets compared to motor vehicle streets with the dominance of green spaces require further research. Given the current tendency in urban centers toward constructing pedestrian streets which usually impose high costs on cities, it is necessary to focus on evaluating the effect of pedestrian streets on mental health compared with other street types. Our findings could motivate urban designers and planners to control environmental factors and conditions and improve the quality of public urban spaces to enhance people’s mental health.

### Limitations and Further Researches

This was a pilot study with no financial support. The small sample size in this study limited the statistical power of our analysis and caused Type II errors (insignificance of the EEG results).

Multichannel EEG is typically considered to be better than the single-channel EEG that we used because it provides more comprehensive information about brain activity. Our evaluations will be more precise and successful if we utilize more data from the five senses. In addition to a multi-channel EEG device, we suggest using a video camera and a voice recorder that record audiovisual data from the subjects during experiments. In this way, the voice, as well as the visual input of the subject, can be constantly monitored. In other words, we can partially perceive the space through the subjects’ point of view and examine the signals on crucial points to measure the effect of environmental factors on the brain.

We should divide the environmental qualities (variables) affecting stress into two categories: acute and chronic stressors. Therefore, various methods must be considered. It is better to design long-term studies to measure the effects of variables, such as land use and physical activity. Based on the separation and link between acute stress/stressors and chronic stress/stressors, future studies can consider the effect of urban space on chronic stress by conducting longitudinal studies for a long time.

We studied three types of streets in one city. After eliminating weaknesses and limitations, we considered replicating the study in a wider range of public urban spaces in different cities to make broader conclusions about the effects of urban environments on stress.

Future research can investigate the effect of different urban spaces, the effect of spaces at different times of the day and night, and the effect of public urban spaces on various cultural groups. There is an important question: Do people from different cultural backgrounds perceive the quality of public urban spaces similarly?

As the effects of urban stress on the nervous system are different in children and adults and people who grow up in large cities are at a greater risk of developing serious health problems in comparison with those who migrate to such cities in their adult life, design of child-friendly spaces entails that future studies address the effect of public urban spaces on children’s mental health.

## Implications for Practice

- Green space is a crucial element of urban design that can effectively reduce stress levels. In land-use planning, the distribution and location of parks and green spaces should be carefully considered to ensure that they are easily accessible to all residents. Furthermore, in the process of subdividing and designing land, the width of streets should be defined with suitable and specific spaces for trees and vegetation cover, which can enhance the overall quality and accessibility of green spaces in urban areas.
- Urban planners and designers should consider designing an integrated network of green boulevards.
- A sense of security is a critical factor in reducing stress levels in urban spaces. To promote this sense of security, urban designers and planners should consider the use of Crime Prevention through Urban Design and Planning tools in the design of urban streets.
- Car-oriented streets cause more stress because of noise pollution, crowdedness, air pollution, and poor pedestrian safety. Urban designers should consider pedestrian-oriented street design and reduce the need for cars by designing an integrated urban transportation network.
